# Contribution of rare whole-genome sequencing variants to plasma protein levels and the missing heritability

**DOI:** 10.1038/s41467-022-30208-8

**Published:** 2022-05-09

**Authors:** Marcin Kierczak, Nima Rafati, Julia Höglund, Hadrien Gourlé, Valeria Lo Faro, Daniel Schmitz, Weronica E. Ek, Ulf Gyllensten, Stefan Enroth, Diana Ekman, Björn Nystedt, Torgny Karlsson, Åsa Johansson

**Affiliations:** 1 Department of Cell and Molecular Biology, National Bioinformatics Infrastructure Sweden, Science for Life Laboratory, Uppsala University, Uppsala, Sweden; 2 Department of Medical Biochemistry and Microbiology, National Bioinformatics Infrastructure Sweden, Science for Life Laboratory, Uppsala University, Uppsala, Sweden; 3 Department of Immunology, Genetics and Pathology, Science for Life Laboratory, Uppsala University, Uppsala, Sweden; 4 Department of Biochemistry and Biophysics, National Bioinformatics Infrastructure Sweden, Science for Life Laboratory, Stockholm University, Stockholm, Sweden

**Keywords:** Biomarkers, Next-generation sequencing, Genome-wide association studies

## Abstract

Despite the success of genome-wide association studies, much of the genetic contribution to complex traits remains unexplained. Here, we analyse high coverage whole-genome sequencing data, to evaluate the contribution of rare genetic variants to 414 plasma proteins. The frequency distribution of genetic variants is skewed towards the rare spectrum, and damaging variants are more often rare. We estimate that less than 4.3% of the narrow-sense heritability is expected to be explained by rare variants in our cohort. Using a gene-based approach, we identify *Cis*-associations for 237 of the proteins, which is slightly more compared to a GWAS (*N* = 213), and we identify 34 associated loci in *Trans*. Several associations are driven by rare variants, which have larger effects, on average. We therefore conclude that rare variants could be of importance for precision medicine applications, but have a more limited contribution to the missing heritability of complex diseases.

## Abstract (web summary)

Despite the success of genome-wide association studies, much of the genetic contribution to complex traits remains unexplained. Here, the authors identify effects by rare variants on plasma proteins, and estimate the contribution of rare variants to the heritability.

## Introduction

Since the advent of genome-wide association studies (GWAS), thousands of genetic variants have been identified to be associated with common diseases and other health-related traits. However, the genetic variants identified to-date explain a limited part of the heritability of most common diseases and traits. For example, in one of the largest GWAS for body mass index (BMI) until now, 941 significant lead single nucleotide polymorphisms (SNPs) explain only 6% of the variation in BMI^1^. By combining the effects of all SNPs together, regardless of whether they reach the genome-wide significance threshold or not, one can capture a substantial part of the hertability of a trait^2^, i.e., SNP heritability, but not all. Here, our working hypothesis is that a part of the complex traits’ heritability can be attributed to the effect of rare genetic variants. These rare variants are not identified as genome-wide significant in GWAS due to lack of statistical power, as only a handful (or less) of individuals, not homozygous for the major allele, may be present in the cohort.

The vast majority of GWAS has been performed on SNP array data, where rare variants are substantially underrepresented. Whole-genome sequencing (WGS) is soon to become a golden standard in large-scale genetic studies, allowing characterisation of genetic variations, such as single nucleotide variants (SNVs) and short insertions and deletions (indels), at any frequency. In natural populations, purifying selection acts against deleterious variants and keeps the frequency of such variants low. Rare variants are therefore more likely to be functionally important than common variants. One example is the melanocortin 4 receptor gene (*MC4R*), where one common allele is associated with a small (~0.25 kg/m^2^) increase in BMI^3^. However, rare deleterious mutations in *MC4R* represent the most common monogenic cause of severe early onset obesity^4^. By performing WGS on more than 1000 Swedish samples, we showed recently that rare variants constitute the major part of all genetic variants in a cohort^5,6^. This supports the need to also analyse the effect of rare variants in relation to complex diseases and traits.

The low frequency of most sequence variants^5^ seriously limits the power of using a GWAS strategy (single-marker tests) for analysing WGS data^6^. In order to overcome these limitations, rare variants can be collapsed and analysed jointly in a burden test^7–9^. By filtering on the predicted deleteriousness of rare variants, and including only loss of function (LoF) or deleterious variants, various studies have shown that the burden of damaging rare variants is associated with complex traits and diseases^10–13^. Variants can also be weighted by their allele frequency, assuming that rare variants are more pathogenic than the common ones, or be weighted by their predicted deleteriousness^14^. However, while rare variants are more likely to have a functional consequence than common variants, most genetic variants in the genome, including the rare ones, are neutral^15^. Therefore, kernel association methods that do not assume uniform directionality and magnitude of effects of all tested variables, are more appropriate when the data is not limited to LoF variants. One such method, the Sequence Kernel Association Test (SKAT)^14^, modulates the effect of multiple genetic variants together in a multivariable approach.

The majority of studies aiming to investigate the effect of rare variants share the common approach of using a test that collapses the effects of multiple genetic variants. It is also common that a cutoff for minor allele frequency (MAF)^12,16^ is used, sometimes limiting the analysis to only very (ultra) rare^17^ variants, or low-frequency variants^16^. The aim of the current study was instead to identify the contribution of rare genetic variants, on top of the common GWAS variants. In previous studies, we have performed GWAS on the protein abundance levels of hundreds of proteins using genotyped and imputed SNPs. We showed that the levels of many of these proteins have high heritability, with up to 67%^18^, and are strongly influenced by genetic variants commonly located in the *Cis*-regulatory regions of the gene encoding the protein itself. The protein dataset is therefore very well suited for investigating, not only the effect of rare coding variants, but also the effect of regulatory non-coding variants.

In the current study, we are analysing SNVs and indels, identified by high coverage WGS, in relation to the protein expression levels of 414 plasma proteins (Supplementary Data 1). We have analysed the same proteins as in our previous GWAS^6,18–21^, but here we are instead using WGS data in combination with the gene-based SKAT method, to also include and identify the contribution of rare variants to variation in plasma protein levels. We show that many associations are driven by rare variants, but a large fraction of the associations are also detected in the GWAS.

## Results

**Fig. 1:**
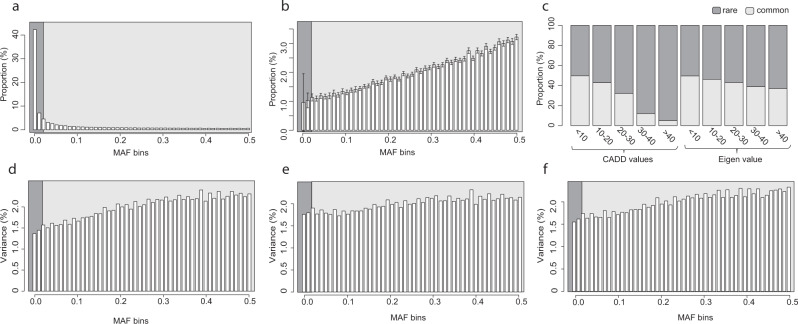
Distribution of MAF, CADD/Eigen values, and fraction of variances across MAF-bins for SNVs and indels. In all figures, the dark grey area indicates the rare variants, as defined in our analyses (MAF < = 0.0239), and the light grey indicates the common variants (MAF > 0.0239). **a** The MAF distribution of the variants identified in the NSPHS. The bars represent the proportion of variants with a MAF within each frequency bin. **b** Distribution of per sample allele counts for different MAF-bins. The bars represent proportion of alleles per sample belonging to different MAF-bins. Averages across all samples (*N* = 1021 with WGS data were used to derive statistics) are shown and the error bars represent the 95% width of the distribution in the cohort. **c** The fraction of the SNVs and indels being rare vs. common, for different CADD and Eigen values. **d** The proportion of genotype variance that can be attributed to variants within the MAF-bins. Each bar represents the sum of all genotype variances for variants with the MAF-bin divided by the sum of genotype variances across all variants. **e**, **f** Proportion of additive genetic variance (narrow-sense heritability) that can be attributed to variants in different MAF-bins when allelic effect sizes are weighted by **e** CADD values and **f** Eigen values.

In total, 872 participants with a median age of 50 years (range: 14–94 years) passed WGS and protein quality control (QC) (Supplementary Fig. 1) out of which 443 (50.8%) were females. In total, 16,271,782 variants were called of which 12,956,981 biallelic SNVs and 1,130,297 biallelic indels passed QC. By using a MAF threshold equal to 1/√(2 x sample size) = 0.0239 as the upper limit for considering a variant to be rare, in agreement with previous suggestions^22^, nearly half (49.4%) of the variants were considered rare. The spectrum of frequencies was highly skewed towards lower frequencies (Fig. 1a).

### Common variants explain most of the heritability

Even if an ample fraction of identified variants were considered rare in the cohort, each individual carries a considerably larger number of common alleles (Fig. 1b). On average, only 2.27 % (standard deviation [SD] = 0.63%) of the variants in each individual were considered being rare in the cohort (dark grey in Fig. 1b). Variants were annotated with Combined Annotation Dependent Depletion (CADD)^23^ and Eigen^24^ scores, commonly used for annotating the pathogenicity of coding and non-coding variants respectively. There was a much larger fraction (*χ*^2^ test; *p* < 2.2 × 10^−16^) of rare variants among the high CADD and high Eigen values (Fig. 1c). For example, while 50% of the variants with low CADD values (< 10) were rare, as much as 95% of the variants with the strongest predicted deleterious effects (CADD > 40) were rare. This agrees with rare variants being more likely to have deleterious effects. To estimate to what extent the rare variants contribute to the narrow-sense heritability in the cohort, we combined (see Methods section) the estimated genotype variance at each SNV and the predicted deleteriousness (CADD or Eigen value). Since each individual carries a considerably larger number of common alleles (Fig. 1b), the genotype variances across all SNVs in the cohort are mainly due to common variants (Fig. 1d). Note that these genotype variances do not take into account that rare variants are more likely to be deleterious (Fig. 1c) and the fractions of explained variance by rare variants (Fig. 1d) could therefore be regarded as the lower boundary for how much of the narrow-sense heritability is explained by rare variants. For example, at least 3.41% of the narrow-sense heritability is likely to be explained by the variants that were considered rare (MAF ≤ 0.0239) in our cohort, and 1.37% by variants with MAF ≤ 0.01. However, even if we take into account that deleterious variants are more likely to be rare and weight the allelic effect by the predicted deleteriousness (CADD or Eigen), the contribution of rare variants to the narrow-sense heritability is still limited (Fig. 1e, f) and only a minor fraction (4.28% or 3.83%) of the heritability could be attributed to rare (MAF < 0.0239) variants, when CADD and Eigen values were used as allelic weights, respectively.

As the sample size in the NSPHS is rather limited (*N* = 872), we extended the heritability calculations to whole-exome sequencing (WES) data from the UK Biobank (UKB) 200 K dataset. We evaluated three subsets consisting of: (a) all unrelated white-British participants (*N* = 148,435), (b) a random selection of 10% of the unrelated white-British participants (*N* = 14,844), and (c) a random selection of 1% of the unrelated white-British participants (*N* = 1484). It was clear, that the fraction of singletons and rare variants is much larger with the increasing sample size, but also much larger in the UKB compared to the NSPHS (Supplementary Fig. 2). This is due to the fact that common variants are likely to be polymorphic also in a small sample size, but a larger sample size and more unrelated participants will display a larger number of rare variants. Furthermore, a much larger fraction of the most damaging SNVs in the UKB was rare (Supplementary Fig. 3), similarly to what was seen in the NSPHS (Fig. 1c). While the proportion of genotype variances attributed to different MAF-bins was similar between the three sample sizes in the UKB (Supplementary Fig. 4a–c), the proportion that was attributed to rare variants was considerably larger compared to the NSPHS (Fig. 1d). The same pattern was seen when allelic effects were weighted by the predicted deleteriousness (CADD), and we estimated that 8.5% of the heritability could be explained by variants with a MAF ≤ 0.01 in the UKB WES, which is considerably higher compared to 1.76% for the WGS data in the NSPHS (Fig. 1e). Since this estimate appears to be independent of sample size (Supplementary Fig. 4d–f), it is possible that the lower estimate in the NSPHS is a result of targeting the whole genome, in contrast to targeting only exons in the UKB data. It is well known that common non-coding variants with small effects are overrepresented among GWAS-hits, whereas exonic variants are more often rare and have larger effects. When filtering on only coding variants in the NSPHS, the distribution of the additive genetic variances was indeed more similar between the NSPHS and the UKB (Supplementary Fig. 5), and with the CADD weighting, we estimate that 4.2% of the heritability among coding variants (compared to 1.76 % for the whole genome) could be explained by variants with MAF ≤ 0.01 in the NSPHS. This fraction is considerably larger, but still about half the amount compared to the fraction in UKB. However, the two cohorts investigated are noticeably different in that the NSPHS consists of participants living in the most northern part of Sweden with participants being more genetically related, whereas the UKB participants included in our dataset represent unrelated United Kingdom citizens from much more widespread areas.

### GWAS results

**Fig. 2:**
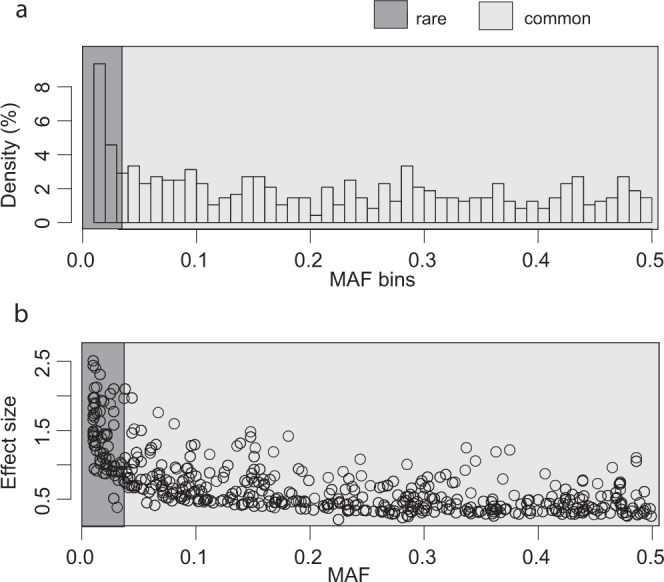
MAFs and effect sizes for the lead GWAS SNVs and indels. **a** Distribution of MAFs for the lead GWAS-significant (Wald-test, *p* < 5.00 × 10^–8^) primary and conditional hits. A MAF threshold of 0.01 was used in the GWAS and, consequently, no GWAS hits had a MAF below 0.01. **b** Effect sizes from the GWAS, in relation to MAF for the primary and conditional GWAS hits. All effect estimates are reported as absolute values.

As a comparison to the SKAT analyses, we performed a traditional GWAS for each protein. However, to identify as many lead GWAS variants as possible in NSPHS, to be adjusted for in the SKAT analyses, we first used a liberal significance threshold of 5 × 10^−8^. A total of 274 proteins had at least one significant SNV or indel (217 proteins had a *Cis* and 107 at least one *Trans*) association (Supplementary Data 2). The lead variants from the GWAS were skewed towards the lower end of the MAF spectrum (Fig. 2a), which agrees with the MAF distribution in the cohort, but is less pronounced (Fig. 1a). However, the power to detect an association drops for rare variants, and with three alleles or less in the NSPHS, the power is zero^6^. Among the lead GWAS variants, the rare variants tend to have larger effect sizes (Fig. 2b) than the common ones, with average beta estimates of 1.52 and 0.62 for rare and common, respectively (*t*-test *p* < 2.2 × 10^−16^). Using stricter *p*-value cutoffs of 3.92 × 10^−11^ for *Trans* and 3.00 × 10^−8^ for *Cis*, which would be more appropriate for identifying novel GWAS hits (Bonferroni adjustment for 414 proteins analysed), resulted in 234 proteins having at least one significant hit of which 212 had associations in *Cis*, and 41 in *Trans*. Four of the proteins with *Trans* hits had associations at more than one locus, and 19 proteins had associations both in *Cis* and *Trans*.

### SKAT analyses

**Fig. 3:**
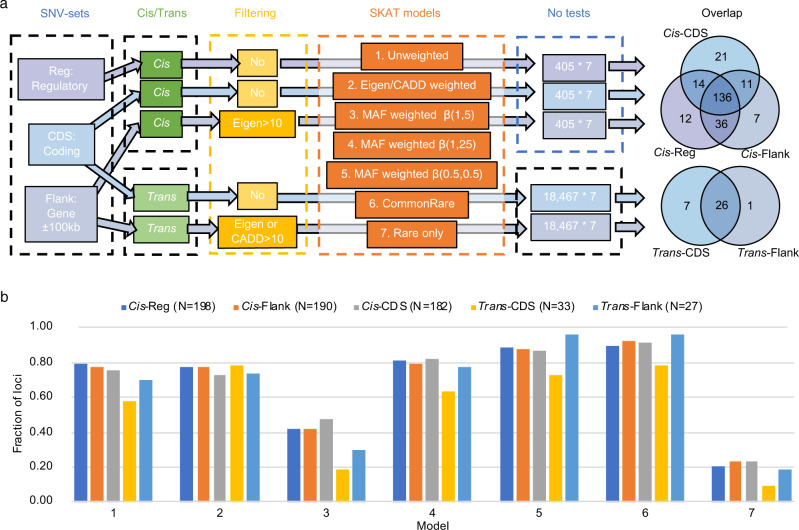
Overview of the SNV-sets, and SKAT tests performed, as well as overlap between the results. **a** Overview of the SNV-sets, SKAT tests performed, and overlap between the results for the different SNV-sets. The Venn diagram shows the number of overlapping loci with any significant SKAT association between the different SNV-sets. For *Cis*-associations a *p*-value of 5.88 × 10^–6^ was considered as threshold for significance, while for *Trans*-associations a threshold *p*-value of 4.67 × 10^–10^ was adopted. **b** Fraction of loci identified in the different models within each SNV-sets. A total of 198, 190, 182, 33, and 27 loci were identified with the five SNV-sets, respectively (*N* in the legend). Each bar represents the fraction of these N loci that were significant for the different SKAT models. The seven models are: (1) Unweighted; (2) CADD or Eigen weighted; (3) MAF weighted, β(1, 25); (4) MAF weighted, β(1, 5); (5) MAF weighted, β(0.5, 0.5); (6) CommonRare; (7) Rare only.

In SKAT, each variant can be assigned a weight that modifies its impact on the test statistics. Commonly, either damaging or rare variants are upweighted assuming they are more likely to be deleterious and the power to detect the effect of a rare variant is typically limited by a small number of observations. In our SKAT analyses (see Methods), five different types of SNV-sets were constructed (Supplementary Fig. 6), and seven different SKAT models, with different weighting and/or filtering of variants based on MAF, CADD or Eigen values, were analysed for each SNV-set (Table 1, Fig. 3a). We considered a MAF threshold of 1/√(2 * sample size) = 0.0239 in our cohort, between common and rare variants as this has been well-motivated in a previous study, and is the default value in the SKAT model^22^.

**Table 1:** Overview and summary statistics for the five types of SNV-sets analysed and the number of significant loci identified with each of the seven SKAT models used.

*Cis/Trans*	SNV-set	No. of SNV-sets^a^	No. of SNVs^b^	Rare^c^	No. significant loci for the different models^d^						
					1	2	3	4	5	6	7
*Cis*	*Cis*-Reg	405	61 [31–116]	47.0%	158	154^g^	83	161	175	178	40
*Cis*	*Cis*-Flank^e^	405	190 [118–307]	48.4%	148	148^g^	80	151	167	176	45
*Cis*	*Cis*-CDS	405	8 [5–13]	49.1%	138	132^h^	87	150	158	167	43
*Trans*	*Trans*-CDS	18,467	8 [5–14]	54.2%	19	26^h^	6	21	24	26	3
*Trans*	*Trans*-Flank^f^	18,467	229 [161–330]	51.3%	19	20^g,h^	8	21	26	26	5

^a^For *Cis*-SNV-sets, each of the 405 autosomal SNV-sets were analysed only in relation to the encoded protein, whereas in the Trans-SNV-sets, the SNV-set (one for each of the 18,467 genes across the genome) was analysed in relation to all 414 proteins. The significance threshold was 0.05/3 *Cis*-sets/405 proteins/seven models = 5.88 × 10^−6^ for *Cis*, and 0.05/2 *Trans*-sets/414 proteins/18,467 SNV-sets/seven models = 4.67 ×  10^−10^ for Trans.

^b^Median [interquartile range] of the number of SNVs in the SNV-sets.

^c^Fraction of SNVs and indels in the SNV-sets that were considered rare (MAF < 0.0239).

^d^The seven models are: model (1) Unweighted, model (2) CADD or Eigen weighted, model (3) MAF weighted—β(1, 25), model (4) MAF weighted—β(1, 5), model (5) MAF weighted—β(0.5, 0.5), model (6) CommonRare, model (7) Rare only, model. See method section for more information on the models and Supplementary Fig. 7 for information on the β-distributions.

^e^Gene ± 100 kb-regions up/downstream of each gene, filtered by Eigen >10 when analysed in *Cis.*

^f^Gene ± 100 kb-regions up/downstream of each gene, filtered by CADD or Eigen >10 when analysed in *Trans*.

^g^Weighted by Eigen values.

^h^Weighted by CADD values.

A total of 237 (59%), out of the 405 proteins encoded by genes located on autosomal chromosomes, were associated (*p* < 5.88 × 10^−6^) with at least one of the *Cis*-SNV-sets (Fig. 3a, Table 1, Supplementary Data 3). Among these, 198 had a significant association with a *Cis-*Reg-set (which includes all variants in regulatory regions in, or close to, the gene), 190 with a *Cis-*Flank-set (which includes all variants in the gene and all variants within 100 kb up and downstream of the gene), and 182 with a *Cis-*CDS-set (including all variants within the coding DNA sequence [CDS] of the gene and variants 40 bps up and downstream of a CDS).

For all SNV-sets, the largest number of significant protein associations was identified using the CommonRare method (model 6) in SKAT, and the lowest number was identified when analysing only rare variants (model 7) closely followed by the model (model 3) where rare variants were highly upweighted (Table 1 and Fig. 3b). It is also worth highlighting that the *Cis*-SKAT analyses weighted by Eigen scores (model 2) did not appear to result in a larger number of significant results compared to no weighting at all (model 1), for any of the SNV-sets (Table 1).

### SKAT *Cis*-associations and backward stepwise regression

**Fig. 4:**
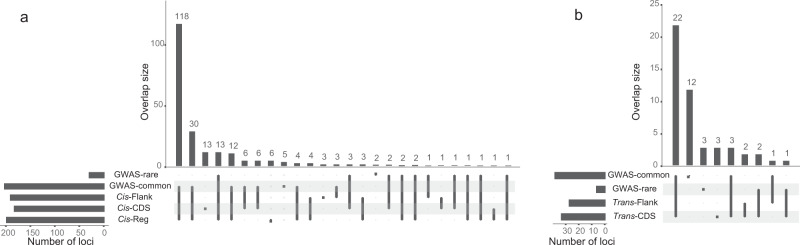
Overlap between the identified loci for the different SNV-sets and the GWAS. The number of loci is the total number of independent loci identified with each SNV-set or GWAS, and the overlap size is the number of loci that overlaps between SNV-sets and GWAS for: **a** *Cis*-associations, and **b** *Trans*-associations. For the *Cis*-associations, a *p*-value (SKAT-test) of 5.88 × 10^‑6^ was considered as threshold for significance for the SKAT analyses, and a *p*-value (Wald-test) of 3.00 × 10^−8^ for the GWAS. For *Trans*-associations, a *p*-value (SKAT-test) of 4.67 × 10^−10^ was considered as threshold for significance for the SKAT analyses, and a *p*-value (Wald-test) of 3.92 × 10^−11^ for the GWAS.

The overlap between the GWAS and the SKAT analyses was very large, with 206 proteins having significant *Cis*-associations detected by both methods. The GWAS identified significant *Cis*-associations with seven proteins (CD4, COL1A1, CCL4, FURIN, IL-18BP, IL-1RA, and WIF-1), for which there were no significant results in any of the SKAT-analyses. In contrast, there were 31 proteins with significant results in the SKAT analyses that were not identified in the GWAS (Fig. 4a and Supplementary Data 3). For 97 proteins, the most significant *p*-value stemmed from a test where rare variants were upweighted (model 3–5). The majority (*N* = 81) of these proteins were associated with a common SNV in the GWAS and when adjusting for these common GWAS SNVs, only 21 of the 81 proteins still had a significant association. This suggests that SKAT methods with rare variants upweighted, to some degree, capture effects by common variants. Among all proteins with overlapping GWAS and SKAT hits, 62 proteins were still significantly associated in the SKAT analyses after adjusting for common GWAS hits (Supplementary Data 3). This indicates that these signals might be driven by rare variants, which was further supported by the fact that 35 (44%) of these proteins were significant also with the SKAT model with rare variants only (model 7). However, other signals could as well have been driven by multiple (common and/or rare) variants, in addition to the ones that reached genome-wide significance in the GWAS.

To elucidate which of the SKAT associations are potentially driven by multiple variants, we performed backward stepwise regression for a subset of the SKAT hits (see Methods). Here, we included (a) all proteins with a *Cis*-SKAT association but without an association with common GWAS SNVs, and (b) all proteins with a *Cis*-SKAT association that were still significant after adjusting for common GWAS SNVs. In order to reduce the number of SNVs to be tested in each multivariable model, only the most significant of the Reg- and CDS-SNV-sets were analysed, and not the larger flank-sets, leaving a total of 73 proteins used in the analyses. In addition to the SNVs that were in the SNV-sets, the GWAS-significant SNPs were also included in the multivariable analyses, even if they were not part of the original SNV-sets. Out of the SNVs that remained after backward stepwise regression (Supplementary Data 4), 38% were rare. We found an average of 5.5 SNVs per protein, and only for 6 of the 73 proteins analysed, the analyses indicated that the signal was driven by one single variant.

### SKAT *Trans*-associations

In total, 34 *Trans*-associated loci were identified for 31 proteins in the SKAT analyses (Supplementary Data 5), which is a slightly lower number compared to the 41 proteins and 45 loci in the GWAS (Supplementary Data 2). There was a considerable overlap between the associated loci for SKAT and GWAS (Fig. 4b). However, among the 26 *Trans*-SKAT associations (loci) that overlapped with a common GWAS association, only seven remained significantly associated in the SKAT tests after conditioning on the lead GWAS SNVs (Supplementary Data 5). Among the proteins with significant *Trans*-hits, several were associated with SNV-sets at multiple loci and/or several neighbouring SNV-sets within the same loci. A number of the identified loci were pleiotropic, i.e., associated with several of the measured proteins, including, for example, the *ABO* locus.

### Including non-coding regions to detect *Trans*-associations

For *Trans*-SKAT associations, there was a lower number of significant hits for the *Trans-*Flank-sets than for the *Trans-*CDS-sets (Figs. 3a and 4b). Also, there was a much larger difference between the performance of the seven SKAT models for the *Trans*-CDS-sets (Fig. 3b), compared to the *Trans-*Flank-sets. This can possibly be explained by a larger number of variants in the SNV-sets being more likely to capture effects through linkage disequilibrium (LD) with SNVs not included in the SNV-set. This suggests that including SNVs outside of the coding regions in the analyses could, in some cases, increase the power to detect associations, partly due to regulatory regions being targeted. However, if the true causal variants are in the CDS-regions of a gene, the CDS-sets are much more likely to capture such effects since the CDS-sets include a lower number of variants and, consequently, a lower degree of freedom in the statistical test, which increases power.

### Pinpointing causal genes in *Trans*, illustrated by the ABO and TNFRSF10C examples

For some proteins, tens of neighbouring genes were located within each *Trans-*Flank-set, and several partly overlapping *Trans-*Flank-sets (including partly the same genes). Therefore, the potentially causal genes behind these associations were not easy to determine. For example, there is a strong association in the *ABO* region on chromosome 9 for several of the proteins (Supplementary Data 5). The most significant association for CDH5 is to the *Trans*-Flank-set surrounding *OBP2B* (*p* = 6.86 × 10^−48^). However, that region also includes *ABO*, suggesting that the signal can as well be driven by *ABO*. In agreement with this, when restricting the analyses to *Trans-*CDS, *ABO* is still highly significant (*p* = 1.18 × 10^−45^), in contrast to *OBP2B* where the *p*-value drops dramatically (*p* = 2.49 × 10^−13^).

**Fig. 5:**
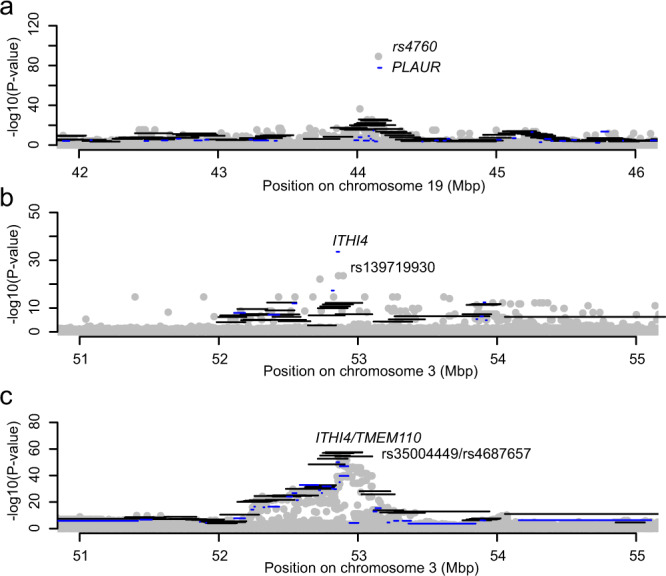
Regional plots for three proteins. The grey circles are the –log10 *p*-values (Wald-test) from the GWAS. Horizontal lines indicate the –log10 *p*-values (SKAT-test) for the *Trans*-Flank-sets in black and *Trans*-CDS-sets in blue. As can be clearly seen, multiple, partly overlapping *Trans*-Flank-sets have been analysed. **a** Results for TNFRSF10C levels, **b** VWC2 levels, and **c** NEP levels.

Another interesting example is a region on chromosome 19, where over 50 SNV-sets are associated with TNFRSF10C levels. While the *p*-values for the *Trans-*Flank-sets are more similar and reach the minimum of *p* = 1.64 × 10^−29^, one *p*-value for the *Trans-*CDS-sets stands out (*p* = 1.64 × 10^−78^). This is the association with the *Trans-*CDS-set for *PLAUR* (Fig. 5a). For this association, it is likely that coding variants in *PLAUR* are driving the association. Indeed, there is also a strong association to a common variant (rs4760, MAF = 0.14, *p* = 6.49 × 10^−90^) for TNFRSF10C identified in the GWAS, and adjusting for this SNV resulted in all SKAT-associations disappearing. The rs4760 is a missense variant that is annotated as deleterious (SIFT) and probably damaging (PolyPhen). Therefore, it is likely that all signals in this region were solely driven by the common variant rs4760.

### *Trans*-association potentially driven by rare variants

Only six of the proteins had any significant *Trans*-association when the analyses were restricted to rare variants (model 7). For three proteins (CCL4, NEP, and TNFRSF10C), the association overlapped with common GWAS SNVs, and the SKAT analyses for these were no longer significant after adjusting for the common SNV. This indicates that the rare-only analyses might also capture effects of common variants through LD. For the other three proteins (Alpha-2-MRAP, PDGF-R-alpha, and VWC2), the associations overlapped with rare GWAS SNVs. One interesting region is the one close to *ITIH4* on chromosome 3. For VWC2, the most significant SNV in the GWAS is a rare variant (MAF = 0.019, *p* = 3.01 × 10^−24^) in this region. However, the SKAT analyses for the rare-only variants in the *Trans-*CDS-set for *ITIH4* resulted in a slightly lower *p*-value (*p* = 2.80 × 10^−27^), which indicates that additional rare variants are driving the signal (Fig. 5b). Interestingly, there are also associations to NEP levels in the same region (Fig. 5c) close to *ITIH4*. Also, for NEP the *Trans-*CDS-sets for *ITIH4* is the most significantly associated (minimum *p* = 1.86 × 10^−50^), but the *p*-value for the rare-only analyses is not even significant (*p* = 0.34). This suggests that the association for NEP levels is most likely driven by a common variant and agrees with the fact that none of the SKAT analyses remained significant after adjusting for rs35004449, the most significant common variant (MAF = 0.36) identified in the GWAS.

### Differences between SKAT weighting models

It appears that several SKAT models as well as SNV-sets performed quite similarly in identifying associations. However, since the largest fraction of the additive genetic variance (narrow-sense heritability) is expected to be explained by common variants (Fig. 1), it is not surprising that the models including common variants with no, or less pronounced, up-weighting of rare variants resulted in the largest number of associations. In fact, the majority of all associations was captured by either model 1, model 2, model 4, model 5, or model 7 (unweighted, CADD/Eigen weighted, and mild up-weighting of rare variants β(1, 5) and β(0.5, 0.5), or CommonRare, respectively). A similar pattern was seen independently on whether only the small SNV-sets (Reg-sets or CDS-sets), or the large Flank-sets were analysed (Supplementary Fig. 8).

**Fig. 6:**
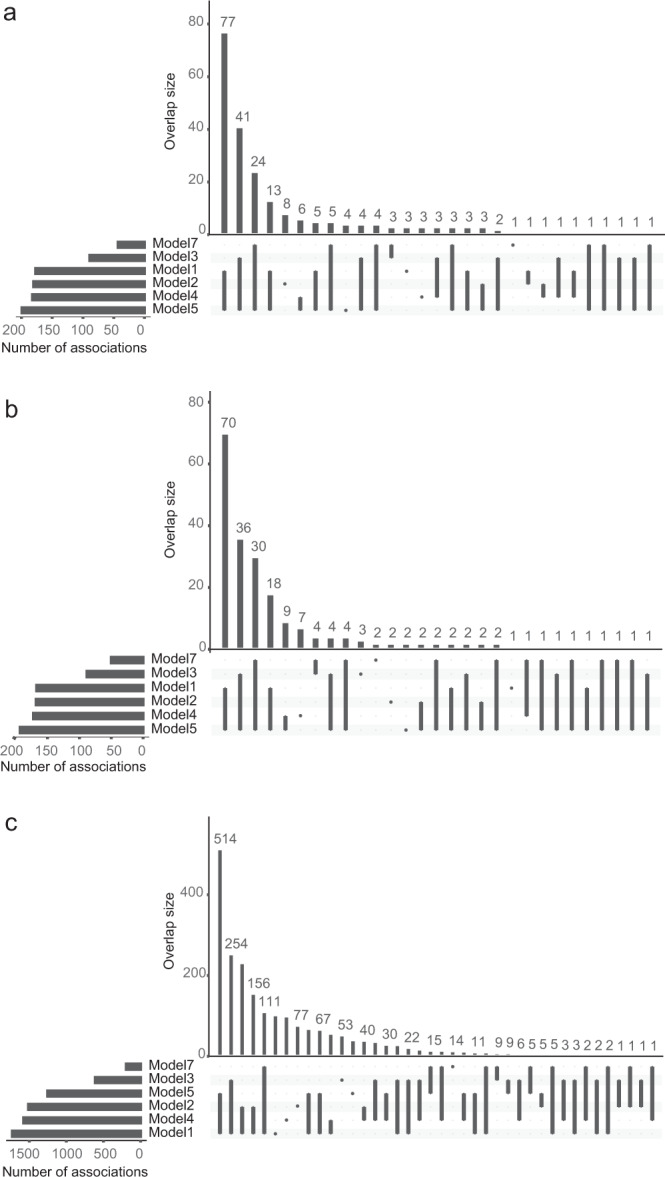
Overlap between the total number of associations for the different SKAT model. The bars to the left represent the total number of associations per model, and the bars in the top (overlap size) the number of associations that overlap between the different models. **a** is the small SNV-sets (CDS-sets and Reg-sets) in the NSPHS, **b** the larger Flank-SNV-sets, and **c** the CDS-sets in the UKB.

To verify that the small difference between models was not solely driven by the small sample size, we performed similar analyses (with CDS-sets) for 27 biomarkers in the UKB WES (see Methods section). The UKB biomarkers were analysed using SAIGE-GENE, where the CommonRare SKAT function is not implemented. In total, 6795 associated CDS-sets were identified (*p* < 1.5 × 10^−8^), that were distributed over 2355 loci (Supplementary Data 6). The pattern of overlap between the associations identified by the six models looked very similar between the NSPHS (Fig. 6a, b) and UKB (Fig. 6c), with the majority of the genes being significantly associated using model 1, model 2, model 4 and model 5, and only a very small subset identified by model 7 (only rare variants).

## Discussion

We have performed a large-scale gene-based association study to evaluate the combined effect of common and rare genetic variants on the expression level of 414 plasma proteins that are either well-established or exploratory biomarkers for different diseases. To some extent, our gene-based results resemble those of a single-marker GWAS, since most of the associations from the gene-based analyses overlapped with GWAS signals, and vice versa. However, for the *Cis*-associations, there was a large number of proteins that only have associatons in the gene-based analyses. This clearly highlights the potential of increasing statistical power of a study by using gene-based tests, which can be partly attributed to the total number of tests being reduced by performing only few tests per gene, instead of one test per genetic variant. In addition, the gene-based analyses can capture effects of multiple variants, where each one of them individually might not reach genome-wide significance in a GWAS.

Gene-based analyses have become more frequent due to increasing availability of large WGS and WES datasets. Until now, the majority of studies have focused on only rare and/or protein-truncating variants from WES data, and utilised burden/variant collapsing tests^10,11,25,26^. Several novel genes have been identified to harbour a burden of rare LoF variants among cases of a disease. A number of gene-based studies using proteomics or other types of omics data have also been performed. For example, using WES data and a large set of metabolites, rare-variant associations were identified for five genes previously known to be associated with the same phenotypes^27^. Few gene-based studies have been performed with WGS data to discover associations with rare variants. For example, Gilly and colleagues^16^ analysed 257 circulating protein biomarkers of cardiometabolic relevance. In agreement with our study, they found a small number of associations to be driven by rare variants. They also highlighted that rare variants are more difficult to replicate between cohorts^16^. Rare variants are more likely to be population specific, and a replication cohort needs to have a similar genetic background. The replication cohort also needs to have a sufficient number of rare effective alleles. However, due to the “winner’s curse”, any statistically significant rare-variant association is likely to contain a larger number of rare effective alleles compared to the population in general. Therefore, a considerably larger replication cohort with similar genetic background is required for replicating rare-variant associations. In our study, we did therefore not replicate the individual rare-variant associations, which should be considered when interpreting results from individual loci. However, we used the UKB WES data and validated the main conclusions of our study.

In contrast to most previous studies, we considered SNVs and indels of any allele frequency as well as any degree of deleteriousness. This enabled us to disentangle associations that are mainly driven by common variants as well as those mainly driven by rare variants, or by a combination of both. Additionally, we also investigated regulatory and gene-flanking variants, which is not possible with WES data. We focused on evaluating and comparing a number of different settings in the SKAT analyses, with regards to weighting, filtering of SNVs and indels, as well as on selecting the SNVs and indels to be included in the SNV-sets. It was clear that different settings are optimal for different genetic architectures. Unsurprisingly, for regions that appeared to be driven by one single-SNV or indel, GWAS appeared to be the most powerful method for identifying the effect, which was indicated by a lower *p*-value. Overall, the SKAT analyses were more powerful than the GWAS since they identified additional signals that did not reach genome-wide significance in the GWAS. Associations that were driven by common variants performed better, i.e., a larger number of associations were identified when either no weighting or mild MAF weighting was applied in the SKAT analyses. Association signals driven by rare variants performed better using a MAF cutoff or by strongly up-weighting rare variants. Since we do not have prior knowledge of the relative contribution of rare and common variants in different regions, it is important to perform different tests to optimise the power to identify genotype-phenotype associations. However, it is also important to bear in mind that increasing the number of tests requires the significance threshold to be adjusted for multiple testing by imposing stricter *p*-value cutoffs. Notably, the analyses where common and rare variants were analysed separately, and the test statistics between these sets were combined (the CommonRare SKAT analyses), outperformed most of the other methods and further method development in that direction would be valuable. Interestingly, the CADD/Eigen weighting did not dramatically influence the results compared to no weighting at all. This may suggest that the values provided by CADD and Eigen are not accurate enough for most variants to be useful in the weighting method, or that the differentiation between harmful and benign variants is too small to render useful as weights.

In agreement with previous studies, we found a relatively low number of association signals for rare variants. This suggests that common variants possibly account for most of the additive genetic variance in a population, i.e., the narrow-sense heritability. This is supported by our calculations showing that the major fraction of the heritability can be attributed to common variants, and that only a small fraction of the heritability is expected to be due to rare variants. This is an important observation that needs to be considered carefully in studies aiming to identify effects by rare variants. However, in agreement with previous studies^10^, we found that the identified effects by the individual rare variants are larger compared to common variants, and we found an enrichment of rare variants among damaging variants. Consequently, the rare variants are likely to play an important role at an individual level, which has also been suggested previously^10^. Rare variants might therefore have a larger contribution to the implementation of precision medicine applications for complex diseases.

We have identified a number of regions that appear to be driven, at least partly, by multiple rare variants, and these associations were further characterised by backward stepwise regression. Interesting examples include AMBP, NGF, CTSZ and CEACAM5, for which the associations were not identified in the GWAS. For both AMBP and NGF, we identified two rare variants with MAF = 0.012 and 0.013 (AMBP) and MAF = 0.0024 and 0.0083 (NGF) as likely drivers of the associations. For CTSZ there are two rare (MAF = 0.0005 and 0.0064) and one low frequency (MAF = 0.045) variant that are likely drivers of the association signal, and for CEACAM5 two rare (MAF = 0.0005 and 0.176) and one common (MAF = 0.419). Another interesting example is ERBB4, for which no significant GWAS SNV was identified, but where 13 SNVs remained after backward stepwise regression. In addition, for some of the proteins where the SKAT associations were still significant after adjusting for GWAS SNVs, a large number of SNVs (both common and rare) remained after the backward regression, including ANPEP, GPC5, and IL5RA. However, it should be noticed that in the backward stepwise regression, no adjustment for multiple testing was performed. These results should therefore not be considered as an estimate of the exact number of contributing SNVs to each signal.

Among the *Trans*-associations, several pleiotropic loci were identified. The *ABO* locus was associated with eight different proteins (CDH5, CTRC, ICAM2, LGALS4, PECAM1, PODXL, SELE, and TEK), but all of these were also identified in the GWAS. The *ABO* gene encodes a galactosyltransferase, which is responsible for the different antigens; A, B, and O, which determine an individual’s blood group. ABO antigens are expressed on red blood cells, but also on epithelial and endothelial cells^28^ and on von Willebrand factor. The association between *ABO* and biomarker expression as well as disease risk has been shown to be very heterogeneous in previous studies. For example, the A antigen has been associated with higher levels of biomarkers related to cell adhesion, the O antigen with lower risk of cardiovascular diseases^29^ and biomarkers reflecting low coagulation activity, and the B antigen increases the risk of type 1 diabetes^30^. Another pleiotropic locus is *ITIH4*, which was associated with both NEP and VWC2. Many neighbouring SNV-sets are associated with NEP levels, which agrees with previous GWAS studies^21^. For both proteins, the most significant SNV-set is the CDS-set for *ITIH4*. However, the NEP association appears to be driven by common variants whereas the VWC2 association appears to be driven by rare variants, which suggests that it is not the same underlying genetic variants that drive the two associations. The most significant GWAS SNV for NEP was also a common variant (rs35004449, MAF = 0.36) in complete LD with a missense variant, rs4687657. However, for VWC2 a rare missense variant (rs139719930, MAF = 0.018) was the most significant. This clearly illustrates that pleiotropic effects at a locus can be due to different genetic effects that possibly act on different pathways. It has previously been suggested that rs4687657 might regulate both the neighbouring genes *ITIH1* and *ITIH4*^31^, so it is possible that the pleiotropic effect observed indeed is due to that the association signals are mediated through different genes.

The SKAT analyses provided a larger number of association signals compared to the GWAS, but also clues towards candidate genes. For example, in the GWAS, five proteins (BMP-6, NRP2, CD38, TNFSF13, and ADAM-TS-15) were associated with common SNVs in the same region on chromosome 5. The lead-SNVs are rs1801020 and rs2545801, which are in high LD (*R*^*2*^ = 0.98), and it can therefore be assumed that their associations are driven by the same underlying genetic effects. The lead-SNVs maps to *GRK6* (G protein-coupled receptor kinase 6). However, in the SKAT analyses, NRP2 is only associated with the *Trans-*CDS-set for *F12*, which is located downstream of *GRK6. F12* encodes a coagulation factor, which makes it the most likely candidate in the region, and here, it is clear that the SKAT analyses perform better than GWAS to highlight candidate genes. This demonstrates the potential to identify candidate genes, by considering only variants in the CDSs, especially when analysing gene-dense regions. However, for *Trans*-associations that are driven by non-coding variants, the ability to identify these associations would drop dramatically if analyses are restricted to CDSs only. Nevertheless, most of these regions included multiple genes, and the underlying causal gene was therefore not easily identified.

It is also important to bear in mind some limitations in our study. First, the antibodies used in the measurements of the protein levels (see Methods section) bind to a short stretch of amino acids in the target protein. Hence, there is a possibility that missense variants are giving rise to altered antibody binding affinity, for example, if a missense variant occurs at the binding site of an antibody, or at a position that creates a protein structure alteration in a way making one antibody unable to bind. Consequently, the target protein could not be detected and quantified. This will appear as if the protein is present in markedly lower levels. In our *Cis*-analyses, we did also consider CDS-sets, even though it is more likely that it is the Reg-sets includes variants that are associated with protein expression levels. Nevertheless, there were as many as 13 associations that were only identified with the CDS-sets (Fig. 4a), which could potentially be caused by missense variants that affect antibody binding. Another limitation was that we only used a gene-centric approach. We did analyse the Flank-sets that contain SNVs and indels located within 100 kb from any gene. These sets are likely to contain most parts of the regulatory regions (Supplementary Fig. 6), but it is possible that additional regulatory elements are located further way from the genes.

In summary, we performed one of the most comprehensive studies to-date, identifying the effect of rare and common genetic variants using WGS data, and we compared several different strategies for gene-based tests. We could clearly show that gene-based tests perform better, especially in regions where multiple rare variants contribute to the effects. However, since we do not know the genetic architecture that contributes to phenotypic variation when designing a study, it is not possible to select one test that will be the best for all regions. Therefore, it is worth highlighting that the CommonRare function in SKAT outperformed the other methods considering the number of identified associations, and also performed reasonably well for associations that are driven by multiple rare variants. Gene-based tests, similarly to GWAS, identify more associations to common than to rare variants. This is partly explained by the fact that a much larger fraction of the phenotypic variance explained is, indeed, due to common rather than to rare variants. Nevertheless, the power to capture effects of rare variants is limited by the low number of observations. Therefore, in future studies of complex traits and diseases substantially larger sample sizes are needed in order to identify effects by rare variants. For genotype-based precision medicine interventions, it is of significant importance to further investigate the impact of rare variants with large effects on one’s individual risk of developing disease.

## Methods

### The Northern Sweden Population Health Study (NSPHS)

The NSPHS (*N* = 1069) was a health survey of the population in the Parishes of Karesuando and Soppero, County of Norrbotten, Sweden^32^. WGS has previously been performed at SciLifeLab in Stockholm, using Illumina short read technology (X-ten) to at least 30x per individual coverage, for the whole cohort following the same pipeline as described previously^5^. The NSPHS was approved by the local ethics committee at the Uppsala University (Regionala Etikprövningsnämnden, Uppsala Dnr 2005:325).

### Quality control (QC) of WGS data

In total, 1041 samples (Supplementary Fig. 1) were sequenced and 20 individuals were removed during variant calling and QC, as has been described previously^6^, leaving 1021 samples for downstream processing. SNV QC was performed using VCFtools (ver. 0.1.13)^33^. Only biallelic SNVs and indels that did not significantly deviate from Hardy–Weinberg equilibrium (*p* > 3.85 × 10^−09^ and *p* > 4.42 × 10^−08^ for SNVs and indels, respectively) were included. We also filtered on genotype quality (GQ > 50), and finally SNVs and indels with >10% missing genotypes were removed. In addition, we removed variants in low-complexity regions based on regions identified previously^34^. VCF-files were converted into binary files and allele frequencies estimated using PLINK v1.90b4.9.

### Protein expression levels

Protein levels for 460 putative biomarkers had previously been measured using the Olink Proseek Multiplex panels (CVD II, CVD III, INF I, ONC II and NEU I, www.olink.com), and the protein extension assay (PEA), as described previously^21^. Briefly, it is an affinity-based assay, where a pair of oligonucleotide-labelled antibody probes bind to the targeted protein. If the two probes are in close proximity, a PCR target sequence is formed, the resulting sequence is detected and then quantified using standard real-time PCR. The samples were analysed on ten different plates with 96-wells each. Of the 96-wells, 92 are samples, one is a negative control and three are positive controls, both used to determine the lower detection limit and to normalise the measurements. We removed proteins with measurements below the detection limit from further analysis. In total, 903 samples were analysed, of which 892 passed the protein QC and 872 passed both protein and WGS QC and were included in the downstream analyses (Supplementary Fig. 1). Protein measurements were adjusted for their position on the plate and standardised using a conservative method where the protein levels for each protein were rank-transformed to be normally distributed (mean = 0, and SD = 1) within each plate. In order to achieve enough power for downstream analyses, only proteins that were above the detection level in at least 400 (46%) of the samples with WGS data were included. Also, two proteins (IL-6 and SCF) had been analysed on two different panels (ONC II and CVD II) and here the ONC II values were removed from the analyses due to lower number of individuals passing QC. After QC, 414 unique proteins remained (Supplementary Data 1) and were analysed in this study.

### Annotation of genetic variant deleteriousness: CADD and Eigen values

Variants were annotated using the CADD (version 1.3, downloaded on 2018-05-14) database to identify what effect the variants are predicted to have on the function of the gene-product. CADD incorporates many different types of annotations and is widely used. From the CADD database, we used the PHRED-scaled CADD scores, meaning that a CADD score above 10 corresponds to the 1% most damaging variants, a CADD score above 20 to the 0.1% most damaging and a CADD score above 30, to the 0.01% most damaging variants^23^. Following this, PHRED-scaled CADD scores can function as a good proxy for functional categories. CADD values were also estimated for indels using the online tool (https://cadd.gs.washington.edu/score, v1.3, March 2018). Variants were also annotated for their predicted effects using Eigen-PC scores v1.1, which is a weighted scoring system commonly used for non-coding variants^24^. Similar to the CADD scores, we used the PHRED-scaled values in all analyses. Individual Eigen-PC scores were available for SNVs but not for indels. However, the Eigen-PC scores are mainly based on the underlying epigenetic pattern for each region^24^ and nearby SNVs have very similar Eigen-PC scores. Therefore, for SNVs and indels with no pre-computed Eigen-PC score available, we used the score of the nearest SNV with pre-computed Eigen-PC score, if such SNVs existed within 100 bp. Finally, we annotated all variants with Ensembl Variant Effect Predictor (VEP, ver. v99.2) with the Loss-Of-Function Transcript Effect Estimator (LOFTEE) plugin^35^, that sorts high-confidence loss of function (LoF) variants from the low-confidence ones. In total, we found 947 high-confidence LoF variants distributed over 827 genes; of which only 84 genes had more than one LoF variant. The low number of LoF variants per gene limits the possibility to perform burden analysis and these annotations were therefore not used in downstream analyses.

### Heritability estimates

We focused only on the narrow-sense heritability: *h*^2^ = *V*_A_/*V*_P_, where *V*_A_ is the additive genetic variance, and *V*_P_ is the phenotypic variance. We further assumed an additive genetic model, which is state-of-the-art in GWAS, with the genotypes coded as dosage values: 0, 1 or 2 copies of the minor allele. The genotype variance at each SNV is simply the variance in the dosage value, var(dosage), across all samples in the cohort. We modelled the variances across genotypes as additive, i.e., all pairwise covariances were set to zero. This is true if we assume that all SNPs with effect on the phenotype are independent. It is a crucial assumption that is commonly used in biological models.

If we assume that all variants have the same phenotypic effect (beta = 1), the sum of the variances across all genotypes, could serve as a measure of the additive genetic variance. This means that: *V*_A_ = Σ 1^2^ * *V*_Ai_ = Σ *V*_Ai_, where *V*_Ai_ = var(dosage) is the variance in dosage of the i-th SNV. However, if we use allelic weights, that correspond to the phenotypic effect of each SNV, the weighted variance at each SNV is instead: var(dosage * weight) = weight^2^ * var(dosage), which means that the variance exhibits quadratic growth in relation to the effect size. In our calculations, we used the CADD or Eigen values as proxies for the phenotypic effects by the variants. Thus, the additive genetic variance becomes: *V*_*A*_ = Σ CADD_i_^2^ * *V*_Ai_, where *V*_Ai_ = var(dosage) denotes the unweighted variance for the i-th SNV, and CADD_i_ is the CADD value for the i-th SNV.

To estimate to the fraction of the variance, across all SNVs in the genome, that is due to rare variants, we estimated *V*_Ai_/*V*_A_, which is the proportion of additive genetic variance that is due to the i-th SNV. Since there is a linear relation between *V*_A_ and *h*^2^ *(h*^2^ = *V*_A_/*V*_P_*), V*_Ai_/*V*_A_ is also the proportion of the heritability that is due to the i-th SNV. The proportion of additive genetic variances across all SNVs in each MAF-bin is therefore a measure of the fraction of the narrow-sense heritability that is explained by the variants in each MAF-bin.

### Annotation of genetic variants to coding, regulatory or gene-flanking regions: CDS-sets, Reg-sets, and Flank-sets

SNVs and indels were first annotated based on whether they belonged to the coding sequence (CDS) of all transcripts in GENCODE (ver. 26). In total, 18,467 genes had at least one SNV or indel that overlapped with any CDSs of its transcripts. For each of these genes, we constructed one SNV-set (referred to as CDS-set), containing all the SNVs and indels that mapped to any of its CDS, or 40 bp up/downstream of a CDS, to include variants important for splicing (Supplementary Fig. 6). Non-coding variants were annotated in relation to potential regulatory regions of the 405 autosomal genes encoding measured proteins. For regulatory regions, transcription starting sites (TSS) and untranslated regions (UTRs) for all possible isoforms of a gene were identified using the GENCODE annotations (ver. 26). A promoter annotation, defined as the region 2 kb upstream of each TSS, was also included. In addition, we selected regulatory regions that overlapped with CTCF binding sites, open chromatin, transcription factor binding sites, promoters, promoter flanking regions and enhancers using Ensembl regulatory annotations (release 92)^36^. A total number of 9674 partly overlapping regulatory regions were identified for 405 autosomal genes encoding the proteins (for example, a promoter annotation as the 2 kb region upstream of a TSS often overlaps partly with the promoter annotation from Ensembl). For each of these genes, all SNVs and indels that mapped to any of its potential regulatory regions, except the 3´UTR, were combined into one regulatory SNV-set (Supplementary Fig. 6) per gene (referred to as Reg-set). SNVs and indels that were annotated to both a CDS-set and a Reg-set were excluded from the Reg-set. The Reg- and CDS-sets above included SNVs and indels that overlapped with the regulatory regions close to a gene, or with the CDSs. However, it is possible that there are regulatory effects that might fall outside these ranges. In order to capture such effects, we also created SNV-sets consisting of all SNVs and indels within 100 kb up/downstream (Supplementary Fig. 6) of each gene (referred to as Flank-set). Here, the start and stop positions (to determine if a variant is within 100 kb up/downstream of the gene) were selected as the min/max coordinate for any of the regulatory regions, or any CDS for respective gene.

### *Cis*- and *Trans*- analyses

The underlying hypothesis, supported by our previous studies^6^, is that the expression of a protein is mainly driven by *Cis*-regulatory genetic variants. In our primary analyses, we therefore analysed both the Reg- and Flank-sets for each of the 405 autosomal genes encoding any of the proteins in (*Cis-*Reg- and *Cis*-Flank-sets). In order to reduce the number of variants tested in the *Cis*-Flank-sets, we included only SNVs and indels with Eigen score > 10. Here, Eigen scores were selected instead of CADD scores, since regulatory variants are more likely to have effect on the expression of a gene in *Cis*. It is also possible that coding variants in a gene, either directly influence the expression level of the encoded protein, or affect the affinity of the antibodies used to measure the protein levels. We could therefore also expect that coding variants in the genes encoding the proteins that had been measured could influence the protein measurements directly (in *Cis*). As sensitivity-analysis, we therefore also tested for associations in *Cis* between the 405 proteins encoded by autosomal genes and the respective CDS-set (*Cis*-CDS-set).

Besides *Cis*-regulatory variants, the expression of a protein can be influenced by *Trans*-regulatory effects. *Trans*-regulatory effects can be mediated through other proteins (or functional RNAs), such as transcription factors, encoded by genes on different chromosomes or located on the same chromosome as the gene encoding the protein itself. *Trans*-regulatory effects can be either due to a coding variant that influence the function of the *Trans*-regulatory protein (or functional RNA), or a regulatory variant that influence the abundance level of the *Trans*-regulatory protein (or functional RNA). For the analyses of *Trans*-associations, we therefore analysed the CDS-sets for each of the 18,467 genes in the genome (*Trans*-CDS-sets). However, Flank-sets were also analysed in *Trans* (*Trans*-Flank-set) for the same 18,467 genes. In order to reduce the number of variants in these *Trans*-Flank-sets the SNVs and indels were included only if CADD > 10 or Eigen > 10, which should capture the 1% most damaging variants with regards to either expression or protein function.

*Trans*-associations were defined as signals that did not overlap with the region surrounding the gene encoding each protein. To exclude all effects of *Cis*-SNVs (due to LD), we required *Trans*-regulatory SNV-sets to be located on a different chromosome as the gene encoding the proteins. Signals located more than 10 Mbp away from the gene on the same chromosome, unless there was a strong *Cis*-association that appeared to extend over more than 10 Mbp, were also considered being *Trans*-associations.

### Statistical analyses

SKAT analyses in NSPHS were performed using SKAT (ver. 2.0.1) in R (ver. 3.5.0). All models were adjusted for sex and age. We included a restricted number of covariates in the models, which is common in GWAS. In previous studies we have shown that several precision variables have strong effects on the levels of some of the proteins investigated^20^. However, it is unlikely that any such variables have an effect on the genetic variants and are therefore not considered being potential confounders in our study. All tests were also adjusted for relatedness by including a pairwise kinship matrix, except for the CommonRare function for which the methodology is not implemented in SKAT. When using the CommonRare function, we instead adjusted for the first 14 genetic principal components. The kinship matrix was constructed using 300,000 autosomal SNPs with MAF > 0.05 selected to represent tagSNPs^6^, and principal components were calculated by first converting the kinship matrix into a distance matrix. The *Trans-*CDS and *Trans-*Flank-sets were analysed in relation to all plasma proteins, which resulted in a total of 18,467 genes times 414 proteins analysed. The *Cis-*CDS, *Cis-*Reg, and *Cis-*Flank-sets were only analysed for autosomal chromosomes and only in relation to the proteins they encoded for, which resulted in 405 gene-protein pairs analysed (Table 1).

All SNV-sets were analysed using the same seven models (Table 1). Our primary SKAT analyses included all variants that had passed QC, independent of MAF, and variants were either unweighted (model 1), weighted by their CADD/Eigen scores (model 2) or by MAF (model 3–5) with three different β-distributions: β(1, 25) where rare variants are dramatically upweighted^37^ compared to common ones that are assigned almost zero weights, β(1, 5) and β(0.5, 0.5) where rare variants are slightly upweighted (Supplementary Fig. 7). We then used the SKAT CommonRare function (model 6), that first analyses common and rare variants separately and then combines the test statistics, using a MAF threshold equal to 1/√(2 * sample size) as cutoff for a variant being considered rare^22^. The sample size differed somewhat for the proteins (Supplementary Data 1), but for the ones that had passed QC in all 872 participants this corresponds to MAF = 0.0239. In the CommonRare analyses, we used the default weights, β(1, 25) for rare and β(0.5, 0.5) for common, where rare variants are weighted against each other with a much higher weight for very rare variants but where the common variants have a much smaller difference in weights between different allele frequencies (Supplementary Fig. 7). Finally, we used a model (model 7) where only the rare variants were considered, using the CommonRare function with test.type argument set to “Rare.Only”. We estimated the family-wise error rate for different analyses by resampling (1000 permutations) to lay between 0.0491 and 0.0504 for the CommonRare analyses and somewhat lower (0.0444–0.0465) for the other SKAT analyses. For the *Cis*-analyses, a Bonferroni-corrected *p*-value of 5.88 × 10^−06^ (0.05/405 proteins/3 *Cis*-SNV-sets/7 SKAT models) was used as threshold for significance (Table 1). For the *Trans*-analyses, the *p*-value threshold for significance was 4.67 × 10^−10^ (0.05/414 proteins/2 types of *Trans*-SNV-sets/18,467 genes/7 SKAT models).

In order to compare the results from our SNV-sets to single-marker association results, and to be able to condition on SNVs identified in a single-marker test, we also performed a separate GWAS for each protein. As in the SKAT analyses, only autosomal chromosomes were analysed. We used the same QC as for SNVs in the primary analyses and the analyses were performed using GEMMA (v. 0.98.1)^37^ with the same covariates (age and sex) and adjustment for relatedness as in the SKAT analyses. In order to capture as many GWAS signals as possible for the conditional analyses, a liberal *p*-value of 5 × 10^−8^ (which is the standard threshold in a GWAS with genotyped/imputed variants) was used as the threshold for significance. For proteins with any single-SNV with a *p*-value below that threshold, conditional analyses were performed in order to identify additional independent single-SNV signals. This procedure was repeated until no additional significant SNV was identified. In the comparisons, with regards to the number of proteins with a significant association between SKAT and the GWAS, a more stringent threshold for multiple testing was considered also in the GWAS. In our previous study, we have estimated the appropriate *p*-value threshold for significance to be 0.05/3,078,707 independent SNVs = 1.62 × 10^−8^ for the WGS data and one phenotype^6^, and the corresponding *p*-value for analysing 414 proteins would therefore be: 1.62 × 10^−8^/414 = 3.92 × 10^−11^ to reach a multiple-testing adjustment, which is as strict as in our SKAT analyses. However, for *Cis*-associations, only 405 proteins and a 2 Mb region up- and downstream of each of the 405 genes encoding the proteins were considered. Therefore, ~405 * 4 Mb = 1616 Mb (about 53.87% of the total genome size), was analysed in total for all proteins together. We therefore adopted a *p*-value threshold of 3.00 × 10^−8^ to fully adjust for multiple testing.

Conditional analyses were then performed using SKAT, adjusting for common GWAS SNVs (both primary and conditional GWAS SNVs). The same threshold for defining a variant as commons vs. rare (MAF = 0.0239), as in the CommonRare analyses, was used. GWAS-significant SNVs with a MAF above the threshold were included as covariates in the SKAT analyses, in addition to the covariates used in the primary analyses. We performed the same set of analyses as above with the same weighting and filtering options. Conditional SKAT analyses were only performed if there was an overlap between the SKAT results and the GWAS results, i.e., if a *Cis*-regulatory SNV/SNV-set was identified with both methods, or if a *Trans*-regulatory SNV/SNV-set in the same region was identified with both methods. Also, from the SKAT analyses, only one SNV-set (with the lowest *p*-value) per locus was included in the conditional analyses, but all seven SKAT methods (Table 1) were used. Here, multiple-testing adjustment was applied, considering the number of proteins analysed and the seven SKAT methods.

Backward stepwise regression was performed in a leave-one-out manner by comparing the likelihoods of two linear, nested models (full and reduced). The full model included all SNVs in a SNV-set, as well as sex, age and PCs. The reduced model included the same variables, except that one randomly selected SNV was excluded. The test statistic was estimated using the *anova* function in R. If there was not a significant difference in the performance of the full and reduced model (*F*-test, *p* > 0.05), the SNV that was excluded in the reduced model was considered as non-contributing to the gene-based association and was excluded from the SNV-set. This procedure was repeated until all SNVs left in the SNV-set were significantly contributing to the model.

### Validation using UK Biobank whole-exome sequencing (WES) data

The UKB includes 502,682 participants from all across the United Kingdom, aged 37-73 at recruitment between 2006 and 2010. The UKB study was approved by the National Research Ethics Committee (REC reference 11/NW/0382). Informed consent to the study was given by all participants. Use of UKB data has been approved by the UKB. The UKB analysis performed in this study has also been approved by the Swedish Ethical Review Authority (dnr: 2020-04415).

Gene-based analyses were performed using the UKB200K WES dataset, in which 200,643 UKB participants have been sequenced. Exome sequencing was performed using IDT xGen Exome Research Panel v1.0 on the Illumina NovaSeq 6000 platform. We excluded first-, and second-degree relatives using kinship data by using a cutoff for an estimated kinship of 0.044. Participants with discordance between self-reported and genetic sex, as well as high heterozygosity, discordance with microarray data or more than 5% missing genotypes were also excluded. Moreover, participants that self-reported not being of white-British descent, or who were not classified as Caucasians by principal component analysis, were excluded resulting in 148,435 participants with WES data included. A total of 4,934,795 polymorphic CDS variants (call rate > 95%) were identified. This is a dramatically larger number compared to the NSPHS. However, reducing the sample size of the UKB cohort, the number of CDS variants decreased accordingly to 1,563,997 variants when including 10% (*N* = 14,844) of the cohort, and 388,570 variants when including 1% of the cohort (*N* = 1484). In order to investigate the MAF distribution and the fraction of variance/heritability that can be attributed rare variants, the three subsets of the UKB cohort (*N* = 148,435; *N* = 14,844; and *N* = 1484) were used in the analyses below. Only autosomal variants were included, and variants were annotated using CADD (version 1.5). The UKB data is reported using hg38 coordinates.

In the UKB, a panel of blood biomarkers were assayed using ten immunoassay analysers (6x DiaSorin Liaison XL & 4x Beckman Coulter DXI 800) and four clinical chemistry analysers (2x Beckman Coulter AU5800 & 2x Siemens Advia 1800). All blood biomarkers were assayed using serum samples, and glycated haemoglobin was measured using red blood cell samples. For details of the assay production please refer to the report at http://biobank.ctsu.ox.ac.uk/showcase/showcase/docs/serum_biochemistry.pdf. For this analysis, we selected a total of 27 biomarkers (apolipoprotein A1, apolipoprotein B, total cholesterol, alanine aminotransferase, aspartate aminotransferase, alkaline phosphatase, calcium, cystatin C, creatinine, C-reactive protein, low-density lipoprotein cholesterol, gamma glutamyltransferase, high-density lipoprotein cholesterol, triglycerides, insulin-like growth factor I, glycated haemoglobin, glucose, sex hormone binding globulin, total protein, urea, phosphate, urate, albumin, direct bilirubin, total bilirubin, and vitamin D) that had been associated with at least one GWAS hit in a previous GWAS^38^, and excluded sex-specific biomarkers (testosterone and 17β-oestradiol).

The gene-based tests were performed using SAIGE-GENE (ver. 0.44.6.5) where the gene-based SKAT-test is implemented and which is better suited for cohorts with larger sample size. The 148,435 unrelated white-British participants were included in the analyses, and since WES data was available, only CDS-sets were analysed. Since most of the biomarkers in the UKB are not protein biomarkers that are encoded by one single gene, all analyses were performed genome-wide. In SAIGE-GENE, the six models were adjusted for sex, age and the first ten principal components. The CommonRare model (model 6) was excluded because it is not implemented in the package. The CDS sets were only analysed for autosomal chromosomes. A Bonferroni-corrected *p*-value of 1.6 × 10^−8^ (0.05/27 biomarkers/18,782 genes/6 models) was used as threshold for significance.

### Reporting summary

Further information on research design is available in the Nature Research Reporting Summary linked to this article.

## Supplementary information

Supplementary Information

Description of Additional Supplementary Files

Supplementary Data 1

Supplementary Data 2

Supplementary Data 3

Supplementary Data 4

Supplementary Data 5

Supplementary Data 6

Reporting Summary

## Data Availability

Pseudonymized individual level data from the UK Biobank, that was used for the current study, are available to bona fide researchers from the UK Biobank, and can be accessed by an application to the UK Biobank. The application process is described in details at the UK Biobank homepage (http://www.ukbiobank.ac.uk/about-biobank-uk/). The UK Biobank data that support the findings of this study are submitted as Supplementary Data 6. The NSPHS dataset analysed in this project are not publicly available due to the sensitive nature of this population and due to the informed consent of the participants. The NSPHS data that support the findings of this study are submitted as Supplementary Data 2–5. Access to pseudonymized individual level data for NSPHS could be granted by the corresponding author (asa.johansson@igp.uu.se) for research purposes. Data access requires that an application has been approved by the Swedish Ethical Review Authority, for which comprehensive information and a digital application portal available at (https://etikprovningsmyndigheten.se/). Briefly, the application must have a clear scientific purpose and clearly describe the objectives, methods, timetable, data management, and ethical considerations, as well as details about the principal investigator and all collaborators that needs to have access to the data, together with information about the entity responsible for the principal investigator. Data delivery is further subject to legal contracts regarding General Data Protection Regulation (GDPR) and Material Transfer Agreement (MTA) between Uppsala University and the receiving entity. Any questions regarding data access could expect a response from the corresponding author within a week. For annotations following databases were used: CADD (version 1.3 and 1.5), Eigen-PC scores v1.1, and GENCODE (ver. 26), and Ensembl regulatory annotations release 92.

## Code availability

Code for the heritability calculations and to reproduce the Fig. 1 is available for download:^39^ https://zenodo.org/record/6380152.
