# Supplementary material for: Contribution of rare whole-genome sequencing variants to plasma protein levels and the missing heritability
Source: Nat Commun. 2022 May 9;13:2532. doi: 10.1038/s41467-022-30208-8 (PMC9085767; doi:10.1038/s41467-022-30208-8)
Supplement: Supplementary file 1 — Supplementary Information [file 41467_2022_30208_MOESM1_ESM.pdf]

Supplementary Information

Contribution of rare whole-genome sequencing variants to plasma protein levels and to the missing heritability

Table of contents:

Supplementary Fig. 1 Flow chart of the study design. 2
Supplementary Fig. 2. Minor allele frequency (MAF) distribution in NSPHS. 3
Supplementary Fig. 3. Fraction of rare variants in different CADD-bins for the three sample sizes. 4
Supplementary Fig. 4. Fraction of the additive genetic variance that is attributed variants in different MAF bin, estimated based on UK Biobank WES data........ 5
Supplementary Fig. 5. Additive genetic variance in NSPHS. 6
Supplementary Fig. 6. Diagram of the SNV-sets. 7
Supplementary Fig. 7. MAF-weights for the variants depending on which parameters are used for the β-distribution........ 8
Supplementary Fig. 8. Overlap between the associations for the different SKAT models........ 9

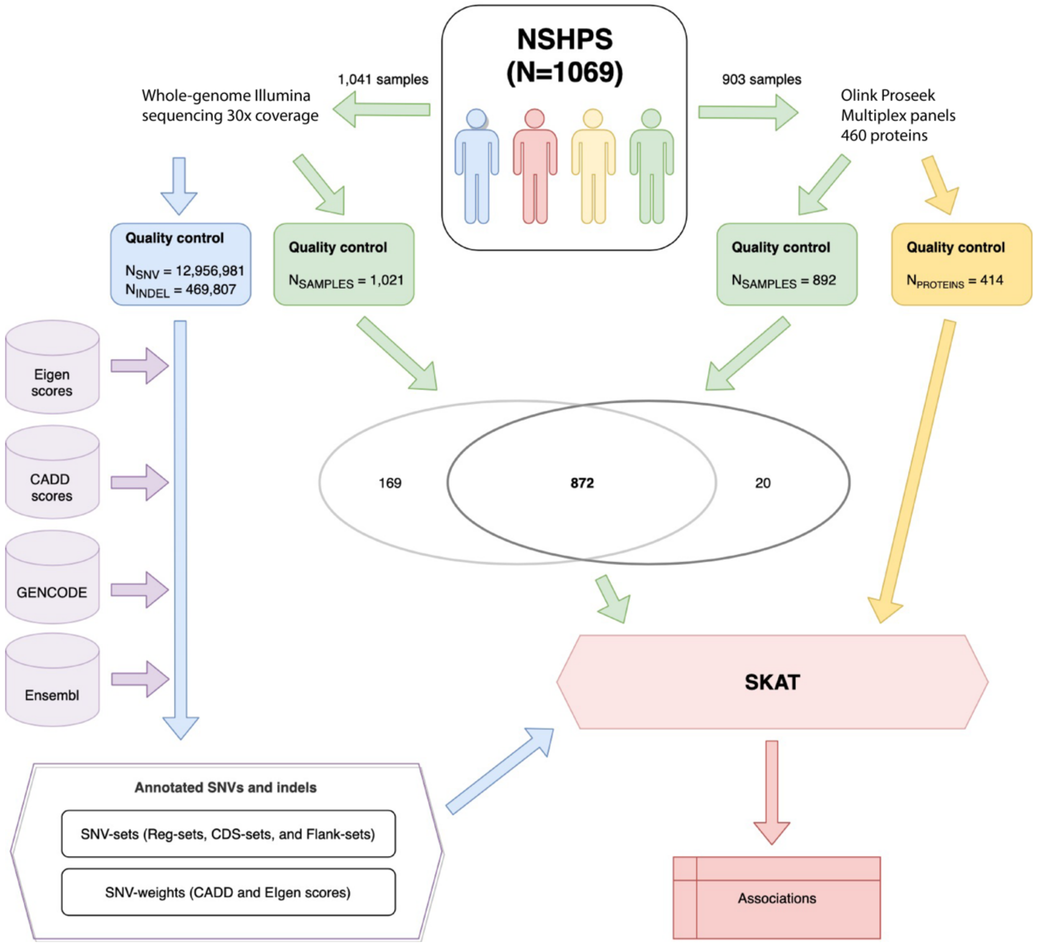

Supplementary Fig. 1 Flow chart of the study design.

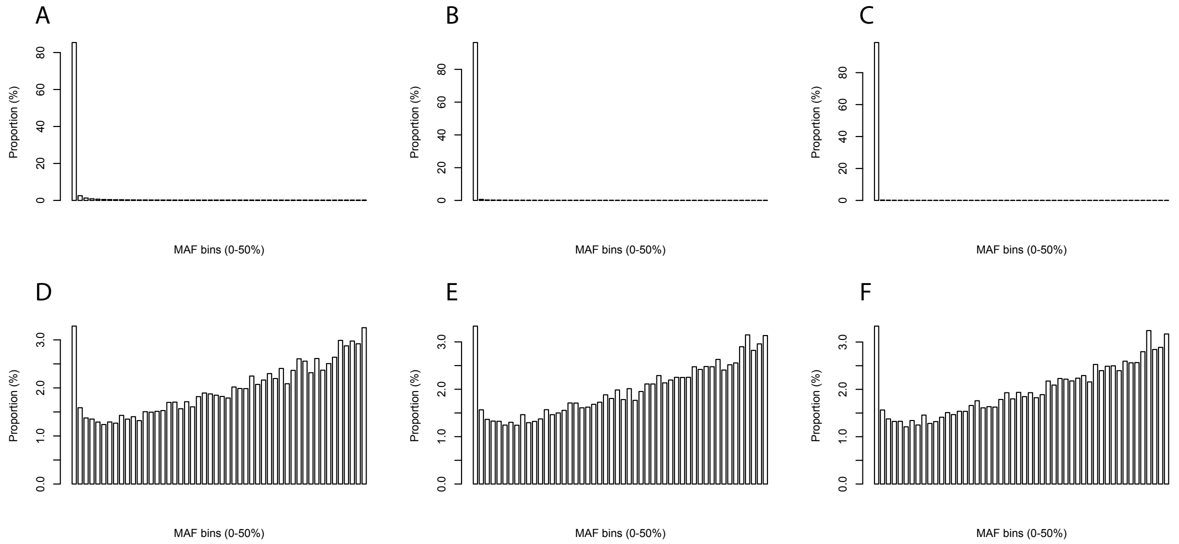

Supplementary Fig. 2. Minor allele frequency (MAF) distribution in NSPHS. A-C) MAF distribution for all polymorphic variants in the three subpopulations with different sample size A) *N* = 1,484, B) *N* = 14,844, and C) *N* = 148,435). D-E) Fraction of allele counts for MAF bin for the three sample sizes D) *N* = 1,484, E) *N* = 14,844, and F) *N* = 148,435). There was a slightly more pronounced skew towards very rare alleles in the larger sub-cohort (C). This was primarily driven by the much larger number (and fraction) of singletons with the larger sample size: 2,066,264 (59.5%) for the largest sample size, 901,352 (57.6%) for mid-sample size, and 230,612 (41.9%) for the small sample size. However, even if a majority of the variants had a MAF below 0.01 (85.4%, 96.4%, and 98.9% respectively with increasing sample size A-C), few individuals carried any of these rare alleles, and only a minority (3.29%, 3.33% and 3.34%) of the total counts of alleles were indeed from low-frequency variants (D-F), with a very similar distribution across sample sizes.

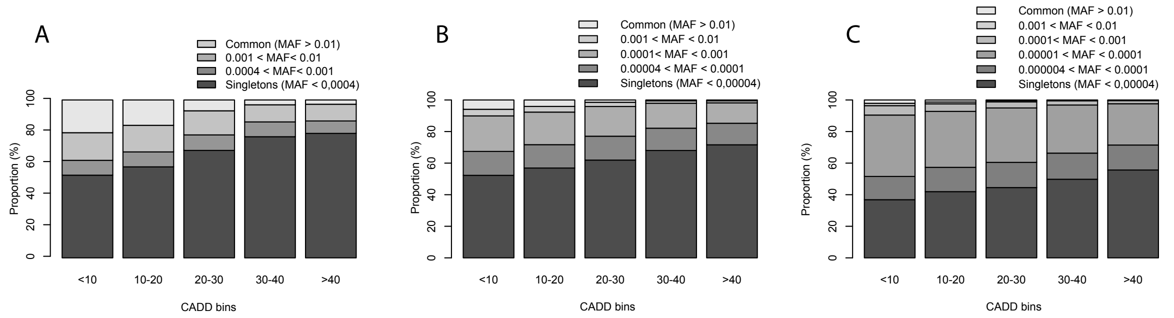

Supplementary Fig. 3. Fraction of rare variants in different CADD-bins for the three sample sizes. A) N = 1,484, B) N = 14,844, and C) N = 148,435). From all three sample sizes, there is a significant enrichment of rare variants (especially singletons) among the most damaging (CADD > 40) class of variants.

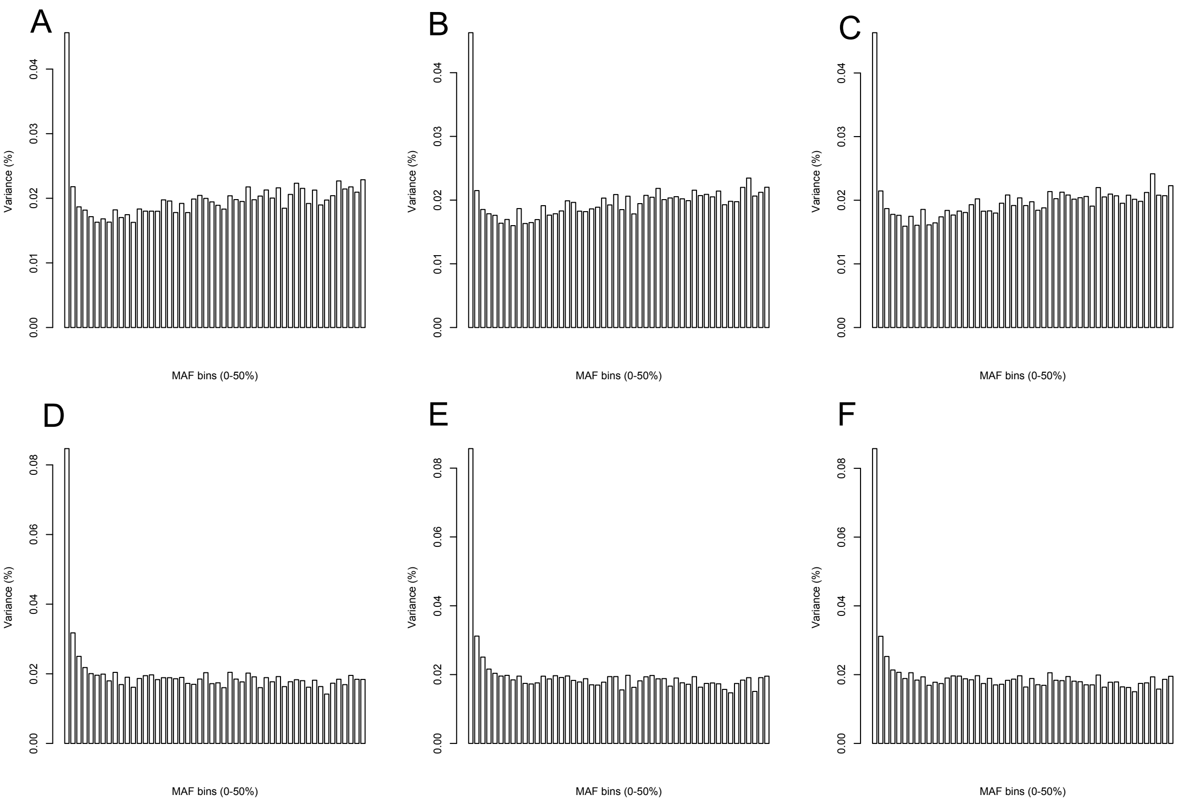

Supplementary Fig. 4. Fraction of the additive genetic variance that is attributed variants in different MAF bin, estimated based on UK Biobank WES data. In A-C, the allelic effects are assumed to be similar between all variants (β = 1), and in D-E, the allelic effects are weighted by the CADD-value of the alleles. The three sample sizes are very similar with A and D) N = 1,484, B and E) N = 14,844, and C and F) N = 148,435).

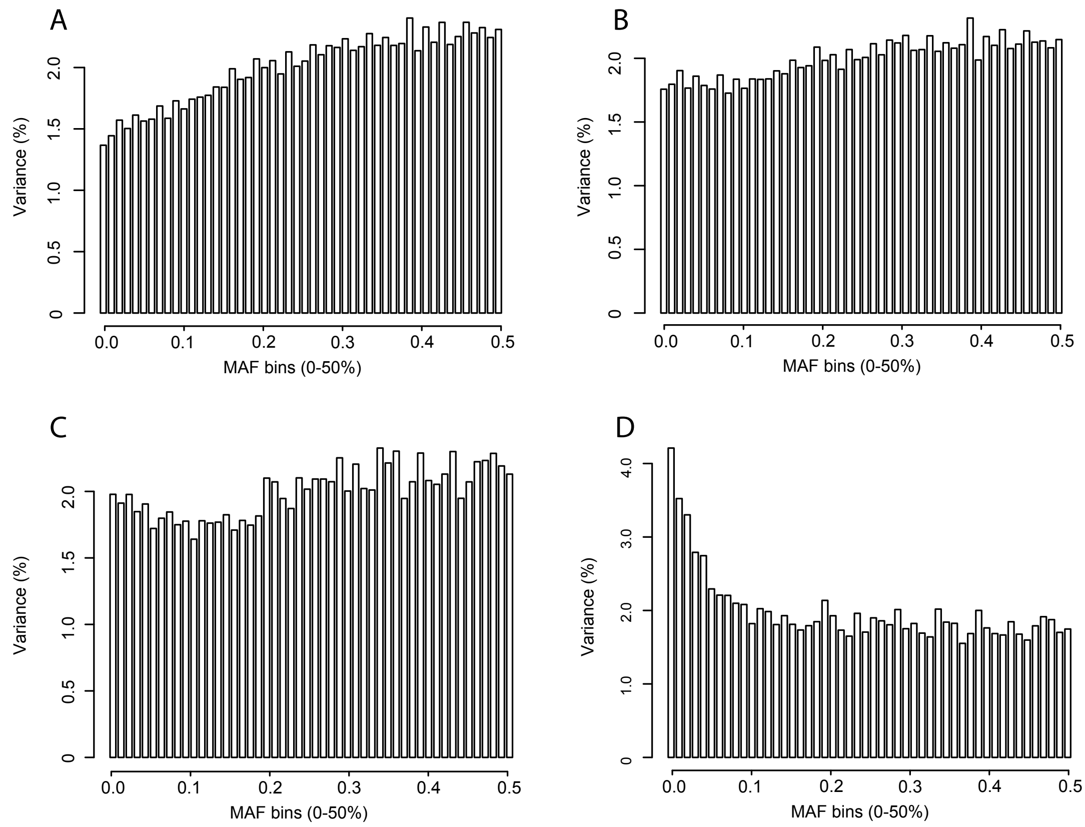

Supplementary Fig. 5. Additive genetic variance in NSPHS. Fraction of the additive genetic variance that is attributed variants in different MAF bin, estimated based on the NSPHS WGS data (A and B), and only the coding variants (C and D). In A and C, the allelic effects are assumed to be similar between all variants (β = 1), and in B-D, the allelic effects are weighted by the CADD-value of the alleles. A and B are the same data as in Figure 1C and 1E.

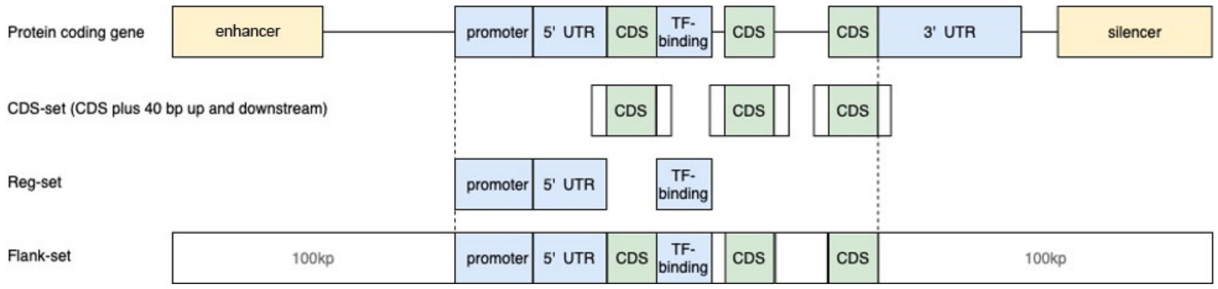

Supplementary Fig. 6. Diagram of the SNV-sets. CDS - Coding sequence, UTR - untranslated region, TF - transcription factor. The CDS-sets contain coding sequenced ±40 bp into the intronic regions to also capture splice sited. Reg-sets contains regions that have been annotated as regulatory and are located in direct proximity to the gene. Flank-sets includes the whole gene-region ±100kb up and downstream of each gene, aiming to also capture more distantly located regulatory regions.

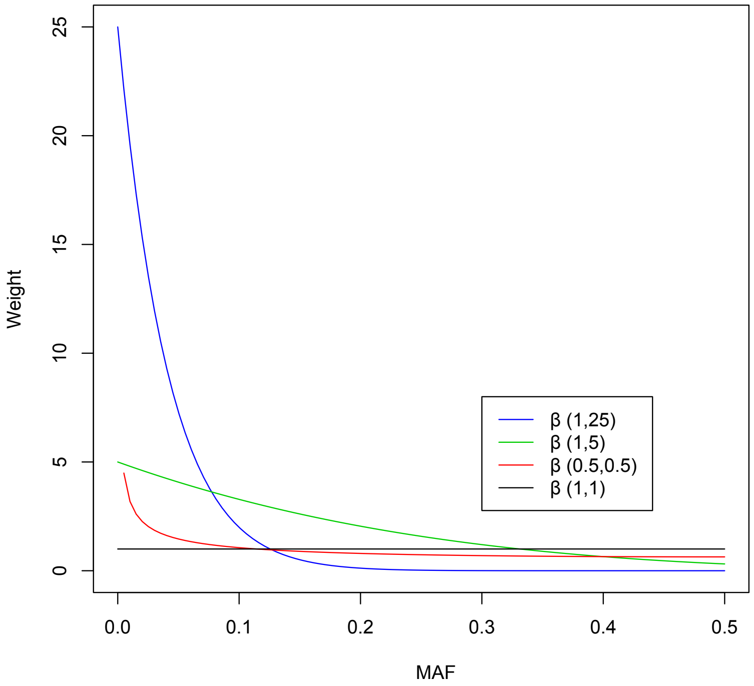

Supplementary Fig. 7. MAF-weights for the variants depending on which parameters are used for the β-distribution. β(1, 1) is without any weights (unweighted) – Model 1 in our SKAT analyses. The other the β-distributions upweights rare variants to different degree and were used in our different SKAT models where, model 3 with β (1, 25) has the strongest up-weighting of rare variants, followed by model 4 with β(1, 5) and model 5 with β(0.5, 0.5). The default values in SKAT are: β(1, 25) and for CommonRare, the default (that we also used in our study) is β(1, 25) for rare and β(0.5, 0.5) for common variants.

A

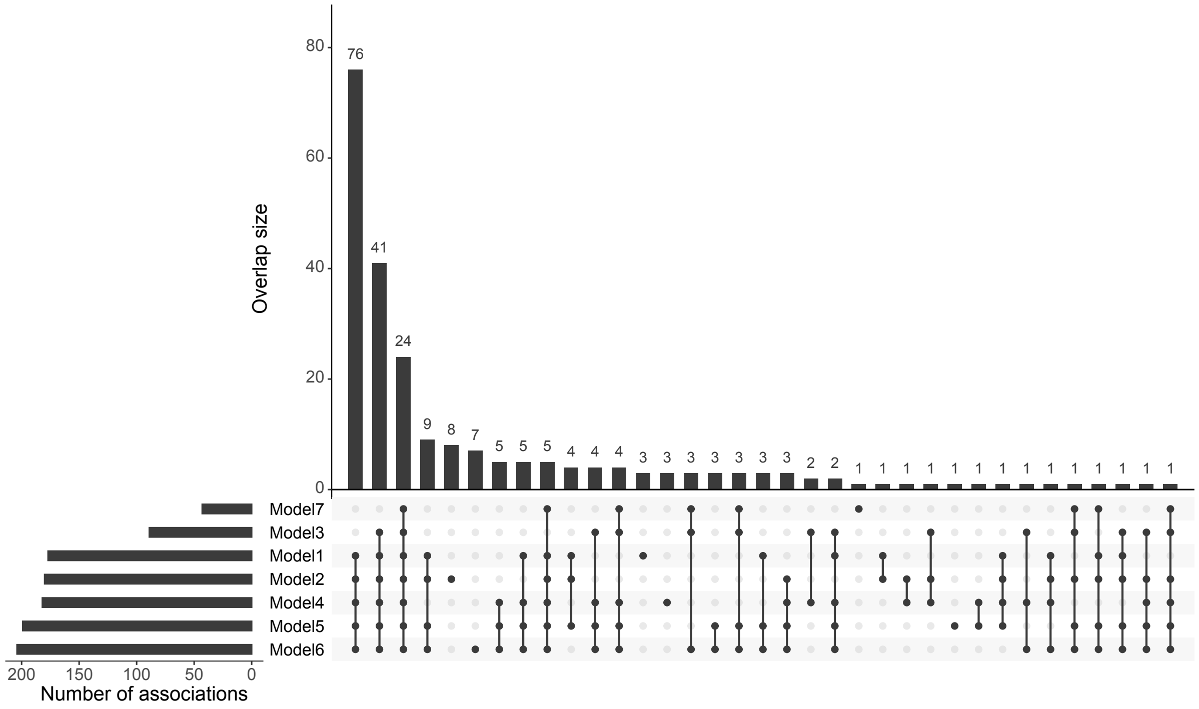

B

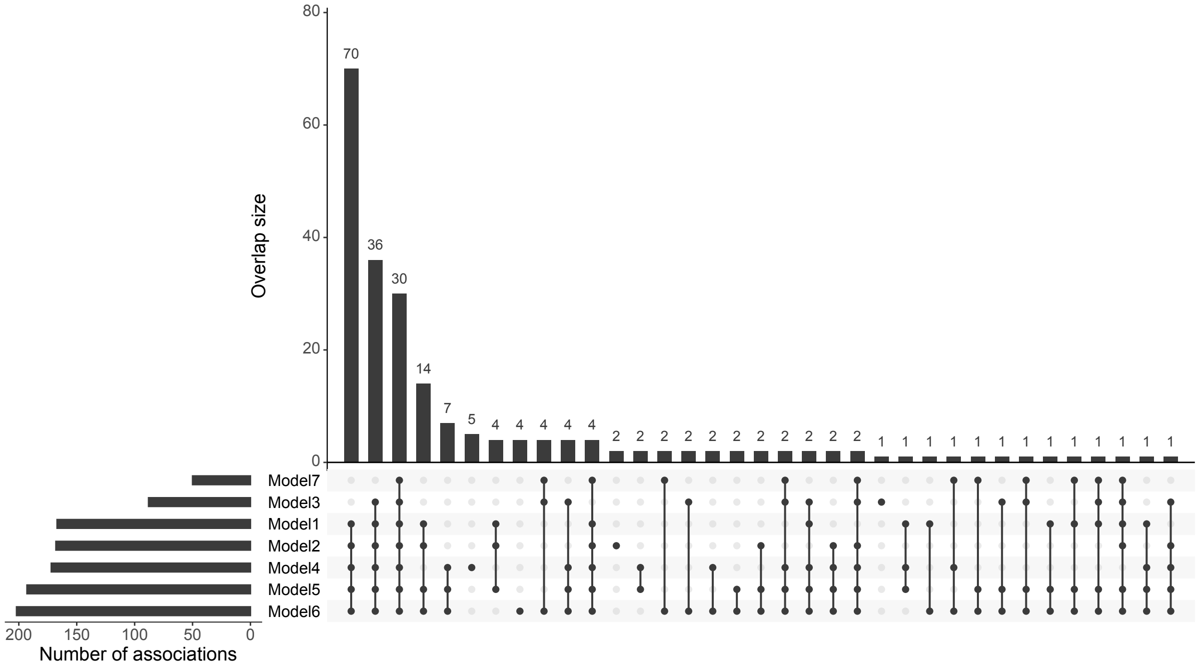

Supplementary Fig. 8. Overlap between the associations for the different SKAT models. The bars to the left represent the total number of associations per model, and the bars in the top (Overlap size) is the number of associations that overlaps between the different models. A) is the small SNV-sets (CDS-sets and Reg-sets), and B) is the larger Flank-SNV-sets.
