# Supplementary material for: Contribution of rare whole-genome sequencing variants to plasma protein levels and the missing heritability
Source: Nat Commun. 2022 May 9;13:2532. doi: 10.1038/s41467-022-30208-8 (PMC9085767; doi:10.1038/s41467-022-30208-8)
Supplement: Supplementary file 2 — Description of Additional Supplementary Files [file 41467_2022_30208_MOESM2_ESM.pdf]

Description of Additional Supplementary Files

File Name: Supplementary Data 1
Description: Information on the proteins analysed

File Name: Supplementary Data 2
Description: Lead GWAS hits from the primary and conditional analyses

File Name: Supplementary Data 3
Description: SKAT results for Cis-associations

File Name: Supplementary Data 4
Description: Multivariable regression for the final model after backward stepwise regression.

File Name: Supplementary Data 5
Description: SKAT results for Trans-associations

File Name: Supplementary Data 6
Description: SKAT results for biomarkers in UK Biobank
