# Supplementary material for: Contribution of rare whole-genome sequencing variants to plasma protein levels and the missing heritability
Source: Nat Commun. 2022 May 9;13:2532. doi: 10.1038/s41467-022-30208-8 (PMC9085767; doi:10.1038/s41467-022-30208-8)
Supplement: Supplementary file 9 — Reporting Summary [file 41467_2022_30208_MOESM9_ESM.pdf]

nature portfolio

Corresponding author(s): Åsa Johansson

Last updated by author(s): Mar 24, 2022

Reporting Summary

Nature Portfolio wishes to improve the reproducibility of the work that we publish. This form provides structure for consistency and transparency in reporting. For further information on Nature Portfolio policies, see our Editorial Policies and the Editorial Policy Checklist.

Statistics

For all statistical analyses, confirm that the following items are present in the figure legend, table legend, main text, or Methods section.

n/a	Confirmed	
☐	☒	The exact sample size (*n*) for each experimental group/condition, given as a discrete number and unit of measurement
☐	☒	A statement on whether measurements were taken from distinct samples or whether the same sample was measured repeatedly
☐	☒	The statistical test(s) used AND whether they are one- or two-sided<br>*Only common tests should be described solely by name; describe more complex techniques in the Methods section.*
☐	☒	A description of all covariates tested
☐	☒	A description of any assumptions or corrections, such as tests of normality and adjustment for multiple comparisons
☐	☒	A full description of the statistical parameters including central tendency (e.g. means) or other basic estimates (e.g. regression coefficient) AND variation (e.g. standard deviation) or associated estimates of uncertainty (e.g. confidence intervals)
☐	☒	For null hypothesis testing, the test statistic (e.g. *F*, *t*, *r*) with confidence intervals, effect sizes, degrees of freedom and *P* value noted<br>*Give P values as exact values whenever suitable.*
☒	☐	For Bayesian analysis, information on the choice of priors and Markov chain Monte Carlo settings
☒	☐	For hierarchical and complex designs, identification of the appropriate level for tests and full reporting of outcomes
☐	☒	Estimates of effect sizes (e.g. Cohen's *d*, Pearson's *r*), indicating how they were calculated

Our web collection on statistics for biologists contains articles on many of the points above.

Software and code

Policy information about availability of computer code

Data collection	no software was used
Data analysis	R version 3.5.0.<br>SKAT: SNP-Set (Sequence) Kernel Association Test. Version: 2.0.1<br>GEMMA: Genome-wide Efficient Mixed Model Association: 0.98.1<br>Scalable and Accurate Implementation of GEneralized mixed model (SAIGE 0.44.6.5)<br>VCFtools (ver. 0.1.13)<br>Ensembl Variant Effect Predictor (VEP, ver. v99.2) with the Loss-Of-Function Transcript Effect Estimator (LOFTEE) plugin<br>PLINK v1.90b4.9<br>Code for the heritability calculations and to reproduce the Figure 1 is available at: https://github.com/AasaJohanssonUU/OpenCode/

For manuscripts utilizing custom algorithms or software that are central to the research but not yet described in published literature, software must be made available to editors and reviewers. We strongly encourage code deposition in a community repository (e.g. GitHub). See the Nature Portfolio guidelines for submitting code & software for further information.

Data

Policy information about availability of data

All manuscripts must include a data availability statement. This statement should provide the following information, where applicable:

- Accession codes, unique identifiers, or web links for publicly available datasets
- A description of any restrictions on data availability
- For clinical datasets or third party data, please ensure that the statement adheres to our policy

Data to support our findings for the UK Biobank are available for bona fide researchers from the UK Biobank (http://www.ukbiobank.ac.uk/about-biobank-uk/), and can be accessed by an application to the UK Biobank. The data from the NSPHS, to support the findings of this study, are available from the corresponding author upon reasonable request and with an ethical approval.
For annotations following databases were used
CADD (version 1.3 and 1.5) database
Eigen-PC scores v1.1
GENCODE (ver. 26)
Ensembl regulatory annotations release 92

Field-specific reporting

Please select the one below that is the best fit for your research. If you are not sure, read the appropriate sections before making your selection.

☒ Life sciences ☐ Behavioural & social sciences ☐ Ecological, evolutionary & environmental sciences

For a reference copy of the document with all sections, see nature.com/documents/nr-reporting-summary-flat.pdf

Life sciences study design

All studies must disclose on these points even when the disclosure is negative.

Sample size	NSPHS, the primary cohort: N=1069. Sample size with WGS and biomarker data that passed QC: 872. We<br>UK Biobank, the secondary study cohort: N=502,682 of which N=148,435 were included in our study.<br>For both cohorts, we included as large sample size as possible that fulfill the inclusion criteria described in the article, and for which we had sequencing and biomarker data available at the time. In these analyses we aimed to use the largest sample available at the time, and for that reason no power calculations were performed.
Data exclusions	Samples for which we did not have access to WGS or WES data or whiteout biomarker data available. In UK Biobank, non-white British participates and related participants were excluded
Replication	The UK Biobank cohort were used for validating the main conclusions from the main analyses (heritability estimations and performance of different SKAT models). In UK Biobank we re-estimated how much of the heritability is due to rare variants and compared and discussed these estimates to the once from NSPHS . We also performed SKAT analyses in UK Biobank using six of the seven SKAT models from the primary analyses, and compared the number of associations identified by each model (Figure 6).<br>The NSPHS have data for a much larger number of biomarkers available so all individual rare variant associations could not be tested for replication.
Randomization	No randomization was done since we are estimating genetic effects and no confounders except for population stratification and relatedness needs to be controlled for. We used the kinship matrix or genetic principal components to adjust for such confounding in our analyses. Covariates (sex, age and batch effects) were also adjusted for the analyses
Blinding	Not applicable. This is an observational study where samples are not stratified by intervention. Blinding was therefore not considered.

Reporting for specific materials, systems and methods

We require information from authors about some types of materials, experimental systems and methods used in many studies. Here, indicate whether each material, system or method listed is relevant to your study. If you are not sure if a list item applies to your research, read the appropriate section before selecting a response.

Materials & experimental systems

n/a	Involved in the study
☒	☐ Antibodies
☒	☐ Eukaryotic cell lines
☒	☐ Palaeontology and archaeology
☒	☐ Animals and other organisms
☒	☐ Human research participants
☒	☐ Clinical data
☒	☐ Dual use research of concern

Methods

n/a	Involved in the study
☒	☐ ChIP-seq
☒	☐ Flow cytometry
☒	☐ MRI-based neuroimaging
